# Integrative Metabolomics, Proteomics and Transcriptomics Analysis Reveals Liver Toxicity of Mesoporous Silica Nanoparticles

**DOI:** 10.3389/fphar.2022.835359

**Published:** 2022-01-27

**Authors:** Jing Li, Runbin Sun, Hui Xu, Guangji Wang

**Affiliations:** ^1^ Lab of Nano-Biology Technology, School of Physics and Electronics, Institute of Super-Microstructure and Ultrafast Process in Advanced Materials, Central South University, Changsha, China; ^2^ Key Laboratory of Drug Metabolism and Pharmacokinetics, State Key Laboratory of Natural Medicines, China Pharmaceutical University, Nanjing, China

**Keywords:** mesoporous silica nanoparticle, metabolomics, proteomics, transcriptomic, hepatotoxicity, oxidative stress, oxidative phosphorylation, inflammation

## Abstract

As pharmaceutical excipients, mesoporous silica nanoparticles (MSNs) have attracted considerable concern based on potential risks to the public. The impact of MSNs on biochemical metabolism is poorly understood, and few studies have compared the effects of MSNs administered via different routes. To evaluate the hepatotoxicity of MSNs, metabolomics, proteomics and transcriptomic analyses were performed in mice after intravenous (20 mg/kg/d) or oral ad-ministration (200 mg/kg/d) of MSNs for 10 days. Intravenous injection induced significant hepatic injury based on pathological inspection and increased the levels of AST/ALT and the inflammatory factors IL-6, IL-1β and TNF-a. Omics data suggested intravenous administration of MSNs perturbed the following metabolites: succinate, hypoxanthine, GSSG, NADP+, NADPH and 6-phosphogluconic acid. In addition, increases in GPX, SOD3, G6PD, HK, and PFK at proteomic and transcriptomic levels suggested elevation of glycolysis and pentose phosphate pathway, synthesis of glutathione and nucleotides, and antioxidative pathway activity, whereas oxidative phosphorylation, TCA and mitochondrial energy metabolism were reduced. On the other hand, oral administration of MSNs disturbed inflammatory factors and metabolites of ribose-5-phosphate, 6-phosphogluconate, GSSG, and NADP+ associated with the pentose phosphate pathway, glutathione synthesis and oxidative stress albeit to a lesser extent than intravenous injection despite the administration of a ten-fold greater dose. Overall, systematic biological data suggested that intravenous injection of nanoparticles of pharmaceutical excipients substantially affected hepatic metabolism function and induced oxidative stress and inflammation, whereas oral administration exhibited milder effects compared with intravenous injection.

## 1 Introduction

Mesoporous silica nanoparticles (MSNs) have been widely used in biology (Rosenholm et al., 2016) and medicine (Tang et al., 2012) due to their high pore volume, large specific surface area, easy surface modification, biocompatibility, and degradability features, such as slow drug release (He et al., 2016; Song et al., 2016). MSNs have been used in cancer treatment (Baeza et al., 2015; Birault et al., 2020), biological diagnosis (Lee et al., 2013), and imaging (Trewyn et al., 2007). Although MSNs exhibit some special, ideal properties as traditional preparation excipient materials, including both degradable or nondegradable produces, these materials are generally considered inert and harmless to the body. However, an increasing number of studies have demonstrated that these tiny nanoparticles can affect the tissues and cells of the body, causing inflammation and histopathological changes (Liu et al., 2012; Peeters et al., 2013; Nemmar et al., 2016; Zhang et al., 2018). Therefore, the assessment of their safety *in vivo* becomes indispensable.

Nanomaterials and porous adsorption particles for pharmacies are generally prepared as injections, oral dosage forms or powder sprays, which enter the body through intravenous, gavage or atomization, respectively (Fu et al., 2013). Nanoparticles easily enter the body through injection and impact the body (Cho et al., 2009; Zhao et al., 2012; Nemmar et al., 2016). In addition, studies have suggested that after orally administered nanoparticles enter the intestine, these nanoparticles can further pass through the intestinal membrane barrier, enter into the circulation system and subsequently tissues and cells, and thus impact the body (He et al., 2011; Li et al., 2015; Chen et al., 2019, 2020). Similar to the processes of solid particles in the atmosphere and in sprays, these solid particles mainly enter the lungs through breathing via the respiratory tract, adhere to the oral cavity and nasal cavity, enter the gastrointestinal tract through drinking or eating, or enter the cells in the oral mucosa of the nasal cavity (Griese, 1999; Patton and Byron, 2007; Park et al., 2010; Garbuzenko et al., 2014; Shin et al., 2017; Pietroiusti et al., 2018; Rosário et al., 2021).

The liver bears the brunt as the target organ of the particles. Lu et al. (2010), (2015) reported that intravenous injection (IV) of silica nanoparticles showed significant capture of the particles in the liver and induced hepatic injury. Huang et al. (2011), Li et al. (2015) administered MSN (particle size over 100 nm) suspensions to ICR mice at a dose of 20 mg/kg by intravenous injection and found increased accumulation in the liver after 7 days. Moreover, oral administration (IG) of MSN suspensions to ICR mice at a dose of 40 mg/kg and spherical MSNs yielded high liver accumulation after 7 days. Mohamed et al. found that acute and subacute oral administration of graphene oxide nanoparticles induced genomic instability and mutagenicity in the mouse liver (Mohamed et al., 2020), which are closely involved in oxidative stress.

Despite sporadic reports on the effects of MSNs on the body, tissues or organs, there is a lack of systematic evaluation on the metabolism, gene and protein levels of the damage to the body, tissues or organs under the conditions of two different dosage forms based on different administration modes, namely injection and oral administration. Considering that the liver is the key primary organ responsible for systematic metabolism and the turnover of small molecules, metabolomics combined with transcriptomics and proteomics was used in this study to assess the effects of intravenous and intragastric administration of MSNs on the function of the liver and to further clarify the effects of administration mode on the body and system. We aim to provide insight into improved assessment of the safety of intravenous injection or oral administration of MSNs and further understanding of the underlying mechanism of hepatotoxicity.

## 2 Materials and Methods

### 2.1 Fabrication and Characterization of Mesoporous Silica Nanoparticles

Mesoporous silica nanoparticles were synthesized according to our previous reports (He et al., 2014, 2017). Briefly, N-octadecyltrimethoxysilanem (C18TMS, 95%) and tetraethyl orthosilicate (TEOS, 98%) were mixed in 50 ml ethanol and sonicated for 10 min. Then, the mixture was transferred into solutions of ethanol, ammonia and deionized water with stirring for 2 h at room temperature. The C18TMS-incorporated particles were collected by filtration, washed with ethanol and deionized water and dried at room temperature. Finally, the collections were calcined at 550°C for 6 h. The morphology and structure of MSNs were observed by scanning electron microscopy (SEM, Hitachi S-4800) and electron microscopy (TEM, JEM-2100F). The surface area, pore volume and pore diameter were calculated by Brunauer-Emmett-Teller (BET) and Barrett-Joyner-Halenda (BJH) methods using a Quantachrome Autosorb-1C apparatus.

### 2.2 Animal Administration and Sample Collection

All animal experiments were performed in accordance with Institutional Animal Care and Use Committee guidelines of China Pharmaceutical University. Male ICR mice (5–7 weeks of age, 20–25 g) were used in our experiments and were purchased from Shanghai Xipu-Bikai Experimental Animal Co., Ltd. All animals were kept on a 12-h light-dark cycle, fed ad libitum and acclimated to our research environment at least 1 week before the experimental manipulation.

Thirty healthy ICR mice were randomly allocated into three groups (*n* = 10 for each group): the control group, IV group and IG group. The MSN suspension (in physiological saline solution) at concentrations of 0.6 mg/ml and 6 mg/ml was ultrasonicated for 20 min before experiments. The suspension was injected through the tail vein (20 mg/kg/d) or administered by gavage (200 mg/kg/d) every day. The treatment period was 10 days.

After fasting overnight on the tenth day, blood samples were collected via the ocular vein, centrifuged twice at 3,000 rpm for 10 min to separate serum and stored at −80°C. After blood collection, the mice were sacrificed immediately. The livers were separated, rinsed in cold phosphate buffer solution, and filter paper was used to soak up water. The hepatic lobes were split into two parts for GC-MS and LC-MS metabolomics. One portion of hepatic lobules was collected for RT–PCR, and the other portion was fixed in 10% formalin for histopathological examination. During the sampling process, all but blood and pathology samples were immediately placed in dry ice and stored at −80°C.

Twenty-four healthy ICR mice were randomly allocated into three groups (*n* = 8 for each group): the control group, IV group and IG group. The previous administration procedure was repeated. After 10 days, the mice were sacrificed, the livers were separated, rinsed in cold phosphate buffer solution, and filter paper was used to soak up water. The hepatic lobes were split into two parts for proteomics and transcriptomics, and all samples were immediately placed in dry ice and stored at −80°C.

### 2.3 Metabolomics Analysis

#### 2.3.1 GC-MS Analysis

The liver samples were pretreated for GC-MS or LC-MS analysis as reported previously (Aa et al., 2021). In brief, 900 μl methanol solution containing [1,2-^13^C_2_] myristic acid (12.5 μg/ml) as the internal standard (IS) was added to 20 mg of liver tissue samples (*n* = 8–9 for each group) in a 1.5-ml Eppendorf tube. The samples were homogenized to precipitate the protein and extract the metabolites. After centrifugation for 10 min at 4°C at 20000×g, 100 μl supernatant was transferred to a chromatography (GC) vial and evaporated to dryness using an SPD2010-230 SpeedVac Concentrator (Thermo Savant, Holbrook, United States). Methoxyamine (30 μl) in pyridine (10 mg/μl) was added to the dried GC vial and shaken to dissolve the metabolites for 5 min. The methoxymation reaction proceeded for 16 h at room temperature. Then, 30 μl of N-methyl-N-(trimethylsilyl) trifluoroacetamide (MSTFA) with 1% TMCS was added to the trimethylsilylation reaction for 1 h. Finally, 30 μl of heptane, including methyl myristate (30 μg/ml), was added to each solution, and the solution was mixed by vortexing for 30 s. GC-MS analysis was performed using a GCMSSQP2010 (Shimadzu Corp., Tokyo, Japan) gas chromatography system, and detailed GCMS parameters are provided in the supporting information. The metabolites were identified by the mass spectra and retention index of the detected compounds with reference standards or those available libraries: the National Institute of Standards and Technology (NIST) library 2.0 (2012), Wiley 9 (Wiley–VCH Verlag GmbH & Co. KGaA, Weinheim, Germany) and our own laboratory at China Pharmaceutical University. Each peak area was normalized based on the internal standard (IS).

#### 2.3.2 LC-MS Analysis

1000 μl methanol-ultrapure water (9:1) containing IS (^13^C-glutamine) was added to 20 mg of liver tissue samples (*n* = 8–9 for each group) in a 1.5-ml Eppendorf tube. The samples were homogenized to precipitate the protein and extract the metabolites. The mixture was centrifuged for 10 min at 4°C at 20000×g. After centrifugation, 400 μl supernatant was transferred to a new Eppendorf tube. The mixture was centrifuged for 10 min at 4°C at 20000×g again. Two hundred microliters of supernatant was transferred to a new Eppendorf tube. Then, the process was repeated. Finally, 100 μl supernatant was collected and added to the chromatography (LC) vial. LC/MS analysis was performed by LC-Q-TOF/MS (AB Sciex), and detailed LC-MS parameters are provided in the supporting information.

Principal component analysis (PCA) and partial least squares discriminant analysis (PLS-DA) were applied using SIMCA-P 14.1. According to the PCA algorithm, each point of the PCA score plot represents the summarized information of all the molecules measured in a single sample. Thus, the distance between points indicates the similarity of metabolic components between samples. PLS-DA can be used to elucidate the separation between groups of variables. MetaboAnalyst3.0 (http://www.metaboanalyst.ca/MetaboAnalyst/) was used to perform KEGG enrichment analysis and generate heatmaps, and the Kyoto Encyclopedia of Genes and Genomes (KEGG) (http://www.genome.jp/kegg/ligand.html) was used to search the related metabolic pathways based on the differential metabolites identified.

### 2.4 Proteomics Analysis

The cold acetone method was used to extract the total proteins. The mouse liver samples (*n* = 5 for each group) were homogenized in SDS protein lysis buffer (8 M urea, 2% SDS, 1x Protease Inhibitor Cocktail (Roche Ltd. Basel, Switzerland)) by vortex oscillation and passage through a high-throughput tissue grinding machine thrice. The supernatant was collected after centrifugation at 12000 g at 4°C for 20 min. The concentrations of the protein extracts were determined using a BCA Protein Assay Kit. After trypsin digestion (Promega, Madison, WI), the peptide mixture was redissolved in 0.1% TFA and fractionated by high pH separation using a Pierce High pH Reversed-Phase Peptide Fractionation Kit (Product No. 84868, Thermo Fisher Scientific, MA, United States). Finally, eight fractions were collected and combined into six fractions; each fraction was dried in a vacuum concentrator for the next step. Then, nano-HPLC–MS/MS analysis was performed by online nanospray LC–MS/MS on an Orbitrap Exploris™ 480 mass spectrometer (Thermo Fisher Scientific, MA, United States) coupled to an EASY-nanoLC 1200 system (Thermo Fisher Scientific, MA, United States). Spectronaut 13 (Biognosys AG, Switzerland) was used to process and analyze the raw DIA data. Proteins were annotated using the GO, KEGG and COG/KOG databases (http://www.geneontology.org) to obtain their functions. After Student’s t test, proteins with a Q value < 0.05 and absolute AVG log2 ratio > 0.58 were filtered as differentially expressed proteins.

### 2.5 Transcriptomics Analysis

Global mRNA was extracted with TRIzol from the mouse liver samples (*n* = 5 for each group) of the control, IV and IG groups. The input material for the RNA sample preparations was a total amount of 2 μg RNA per sample. Sequencing libraries were generated using the NEBNext^®^ Ultra™ RNA Library Prep Kit for Illumina^®^ (#E7530L, NEB, United States) according to the manufacturer’s instructions, and index codes were added to attribute sequences to each sample. Briefly, the mRNA was purified from total RNA by poly-T oligo-attached magnetic beads. Fragmentation was performed using divalent cations under elevated temperature in NEBNext First Strand Synthesis Reaction Buffer (5X). First strand cDNA was synthesized using random hexamer primers and RNase H. Second strand cDNA synthesis was subsequently performed using buffer, dNTPs, DNA polymerase I and RNase H. The library fragment was purified with QiaQuick PCR kits and eluted with EB buffer. Then, terminal repair, A-tailing and adapter addition were implemented. The target products were retrieved, and PCR was performed. Finally, the library was completed. A Qubit^®^ RNA Assay Kit in Qubit^®^ 3.0 was used to measure the RNA concentration of the library to preliminarily quantify and then dilute the sample to 1 ng/μl. An Agilent Bioanalyzer 2100 system (Agilent Technologies, CA, United States) was used to assess insert size, and a StepOnePlus™ Real-Time PCR System (valid library concentration>10 nM) was used to qualify accurate quantification of insert size. Clustering of the index-coded samples was performed on a cBot cluster generation system by HiSeq PE Cluster Kit v4-cBot-HS (Illumina) following the manufacturer’s recommendations. After cluster generation, 150-bp paired-end reads were generated by running a double-ended sequencing program (PE) on the HiSeq sequencing platform. The ENSEMBL database (http://www.ensembl.org/index.html) was used to obtain the reference genomes and the annotation file. HiSeq was used to count each gene in each sample. Genes with a Q value < 0.05 and absolute AVG log2 ratio > 1 were identified as significantly expressed genes.

### 2.6 Biochemical Analysis

Serum biochemistry analyses of alanine aminotransferase (ALT), aspartate aminotransferase (AST), alkaline phosphatase (ALP), albumin (ALB), blood urea nitrogen (BUN), creatinine (CREA) and dehydrogenase (LDH) were performed by Zhongda Hospital Southeast University (Nanjing, China) (*n* = 8 for each group).

### 2.7 Quantitative RT–PCR

Total RNA was isolated from mouse liver samples (*n* = 8 for each group) using TRI Reagent (Sigma, Nanjing). The mRNA concentrations were quantified using a NanoDrop ND-1000 spectrophotometer (Thermo Scientific, Nanjing). The diluted mRNA (0.5 μg/μl) was reverse-transcribed according to the manufacturer’s protocol (Takara Biomedicals, Nanjing), and the gene expression levels were determined by SYBR-green-based real-time-PCR (ABI ViiA 7 Real-time PCR system, Applied Biosystems, United States). β-actin and GAPDH mRNA levels were used for internal normalization. The sequences of TNF-a, IL-6 and IL-1β primers used for qRT–PCR in our study are listed in Supplementary Table S4.

### 2.8 Histological Analysis

Liver samples (*n* = 3) were fixed in formalin for 24 h. Then, the fixed samples were embedded in paraffin and sectioned for histopathology analyses with hematoxylin and eosin (H&E) staining.

### 2.9 Data Analysis and Statistical Analysis

The results are presented as the means ± standard deviation (SD). Statistical analysis was performed by GraphPad Prism 7.0 (GraphPad, San Diego, CA, United States). Unpaired Student’s t-test and one-way ANOVA with Tukey’s correction were used as appropriate. A *p* value < 0.05 was considered statistically significant for all data.

## 3 Results

### 3.1 Physicochemical Properties of the Mesoporous Silica Nanoparticles

Preliminary analysis of the physicochemical properties of the mesoporous silicon nanoparticles was performed using scanning electron microscopy (SEM), transmission electron microscopy (TEM) and nitrogen adsorption-desorption analysis. As shown in the SEM image in Supplementary Figure S1A, the morphology of the MSNs was approximately spherical, and the MSNs exhibited good monodispersity and a uniform particle diameter at approximately 80 nm. Supplementary Figure S1B shows that the materials have worm-like mesostructured. The measured BET specific surface area of the MSNs was 751.193 m^2^/g. The average pore size and pore volume determined by the Barrett–Joyner–Halenda method were 2.76 nm and 0.746 cc/g, respectively (Supplementary Figures S1C,D).

### 3.2 Biochemical, Histopathological and Inflammatory Factors Indicate Hepatic Injury due to Intravenous or Oral Administration of Mesoporous Silica Nanoparticles

**FIGURE 1:**
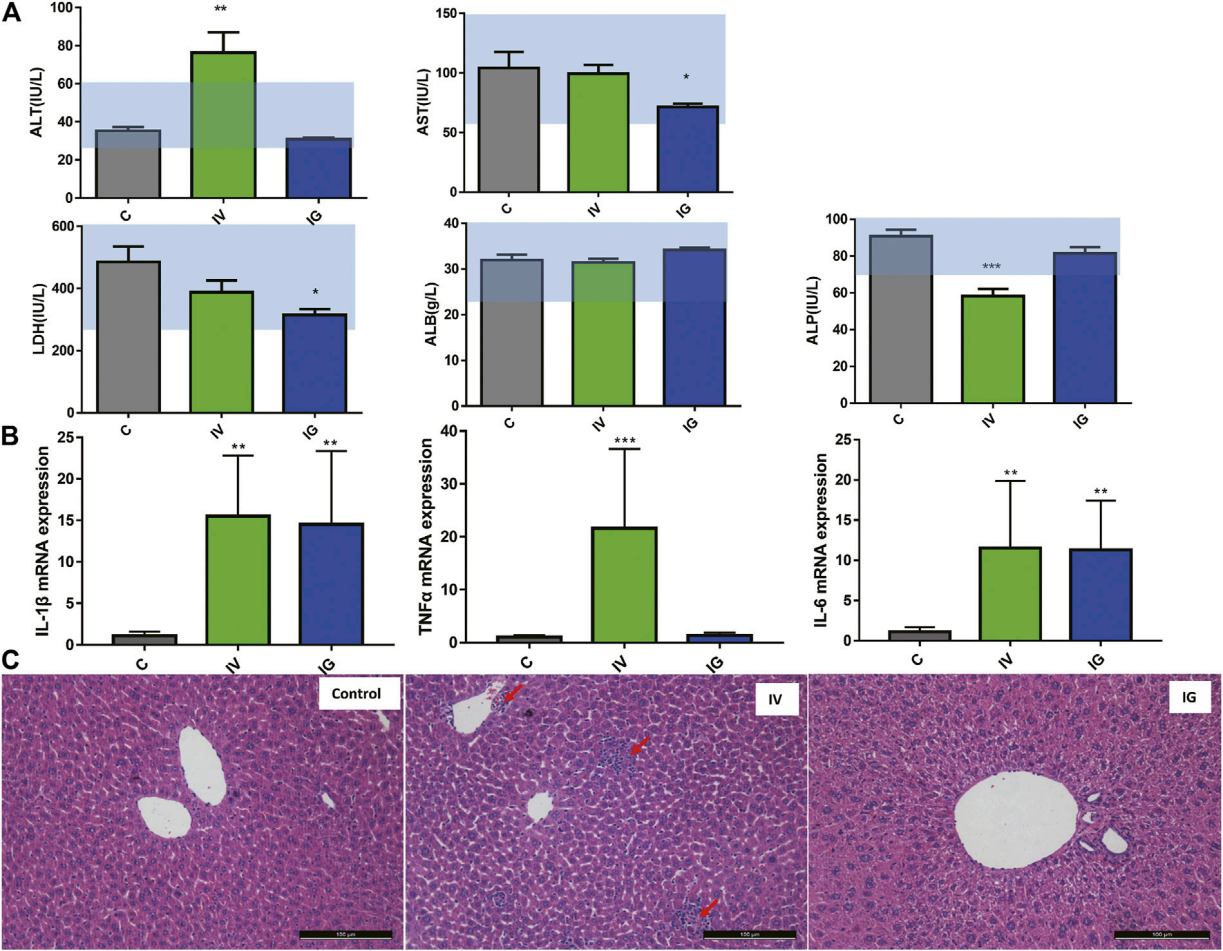
**(A)** Effect of different exposure methods (IV and IG) on serum biochemistry, including ALT, AST, ALP, LDH and ALB. Light blue bars indicate the range of values obtained from healthy ICR mice. **(B)** IL-1b, TNFa and IL-6 mRNA expression in mouse liver. **(C)** Histological examination of liver from the mouse of control group, IV and IG group, red arrow: inflammation site. The data are expressed as the mean ± SD, ∗*p* < 0.05, ∗∗*p* < 0.01, and ∗∗∗*p* < 0.001 compared with the control.

After oral administration or intravenous injection of MSNs for 10 days, the ALT and AST levels were measured to assess hepatic injury. As shown in Supplementary Figures S1A, S2, compared with the control group, oral administration significantly reduced AST, BUN and LDH activities, but these activities were within the normal range. Intravenous injection significantly elevated ALT activities and decreased ALP activities beyond the normal range and downregulated BUN within the normal range. The ALB and CREA concentrations were not changed in the IG and IV groups compared with the control group. Moreover, the mRNA expression of inflammatory cytokines, including IL-1β, IL-6 and TNFα, was measured in the liver tissues of mice. All IL-1β, IL-6 and TNFα expression levels were significantly upregulated in the IV group, and IL-1β and IL-6 expression levels were increased in the IG group compared with the control group (Figure 1B). In addition, representative pathological micrographs of the liver tissue sections are presented (Figure 1C). HE staining pathological images did not show obvious inflammatory cells in the IG group, whereas spotty necrosis accompanied by inflammatory cell infiltration was observed in the IV group (red arrow). Overall, these results indicated that oral administration of MSNs induced a proinflammatory response in the liver and that intravenous injection of MSNs induced significant hepatic injury in mice.

### 3.3 Metabolomics Reveals Metabolic Perturbation in Mouse Liver Exposed to Mesoporous Silica Nanoparticles Administered via the Intravenous or Oral Route

**FIGURE 2:**
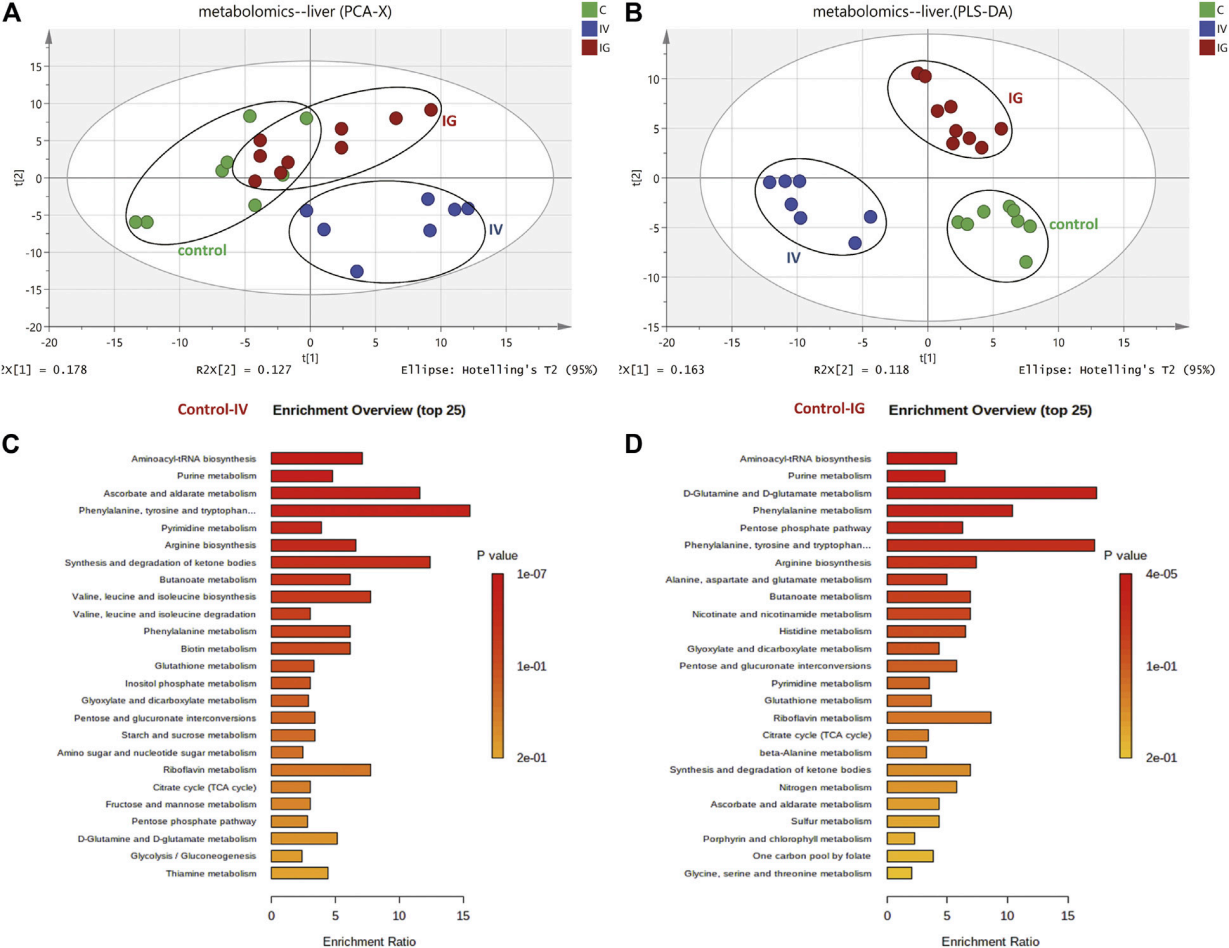
Effect of Mesoporous Silica Nanoparticles (MSNs) on mouse liver metabolites by different exposure methods (IV and IG) using HPLC–MS and GC–MS. **(A)** Principal component analysis (PCA) scoring maps of metabolites. **(B)** Partial least squares discriminant analysis (PLS-DA) scoring maps of metabolites. **(C)** KEGG metabolic pathway of the liver samples between the control and IV groups. **(D)** KEGG metabolic pathway of the liver samples between the control and IG groups.

The metabolomics profiles of the liver samples were analyzed based on a PCA score plot (Figure 2A). The control and IV groups were obviously separated in the PCA score plot, and the control with IG groups exhibited slight separation. Additionally, a three-component PLS-DA model was constructed (Figure 2B). The PLS-DA score plot revealed the goodness of fit and high predictability of the model, demonstrating good separation between the IV group and the control group as well as the IG group and the control group. These PCA and PLS-DA score plot results suggested that intravenous injection with MSNs exhibited remarkable differences and dramatic metabolic disturbances compared to the control group or IG group.

In total, compared to the control group, 18 differentially metabolites was involved in carbohydrate metabolism, 21 differentially was metabolites involved in amino acid and derivative metabolism, 23 differentially metabolites was involved in purine and pyrimidine nucleotide metabolism, 4 differentially metabolites was associated with fatty acid and ketone bodies, 8 differentially metabolites was involved in vitamin and GSH metabolism, and 8 differentially metabolites of others were identified (Supplementary Table S1). Based on KEGG enrichment pathway analysis (Figures 2C,D), the main perturbed metabolic pathways we focused on included the tricarboxylic acid (TCA) cycle, glycolysis, nicotinamide and glutathione metabolism, pentose phosphate pathway (PPP), amino acid metabolism, and purine and pyrimidine nucleotide metabolism.

### 3.4 Correlation Analysis of Transcriptomic and Proteomic Data in Mouse Liver Upon Exposure to Mesoporous Silica Nanoparticles by Intravenous or Oral Administration

**FIGURE 3:**
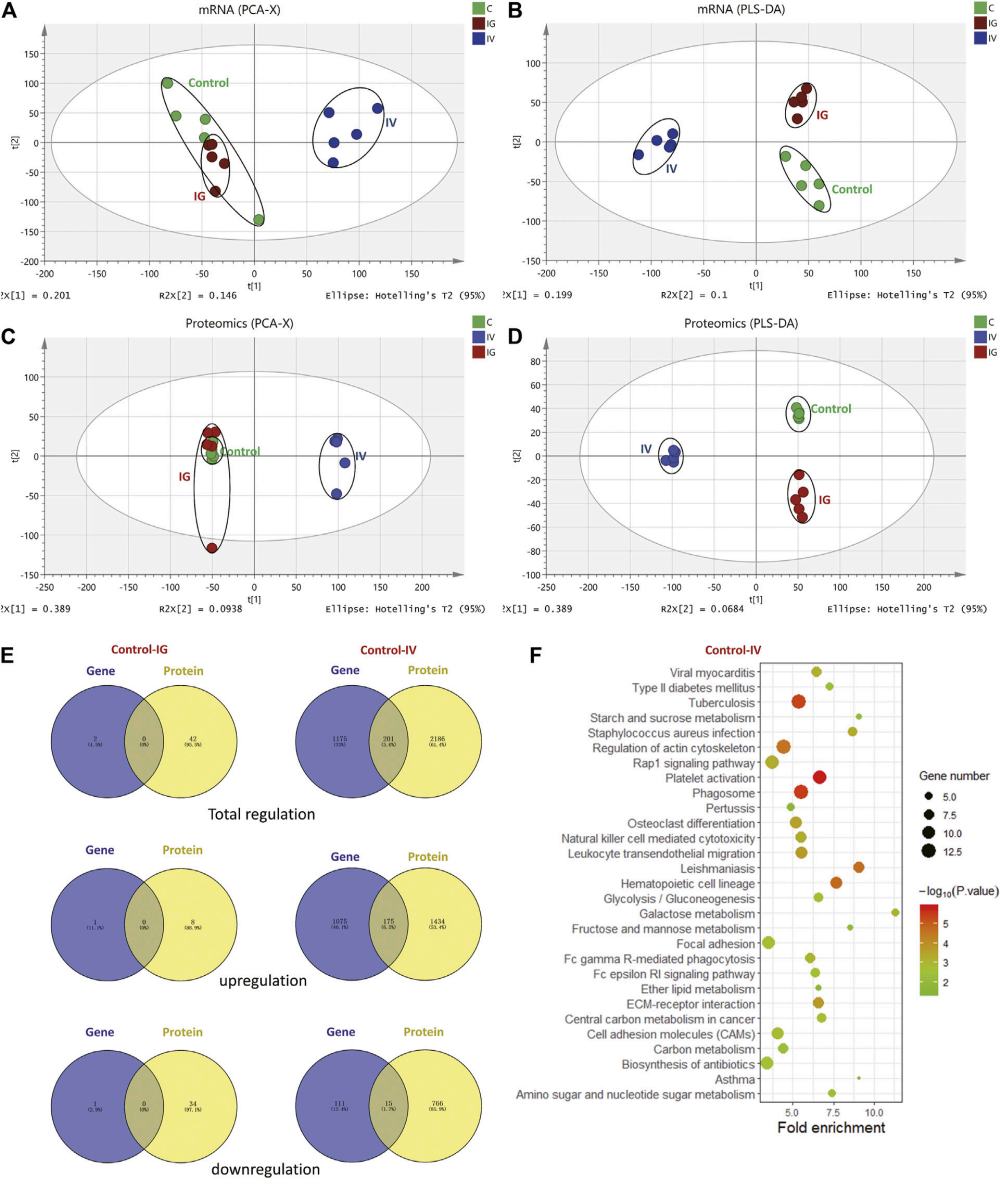
Effect of Mesoporous Silica Nanoparticles (MSNs) administered by different routes (IV and IG) on mouse liver proteins and genes as determined by proteomics and transcriptomics. **(A)** PCA, **(B)** PLS-DA of transcriptomics, **(C)** PCA, **(D)** PLS-DA of proteomics. **(E)** Venn diagrams depict overlapping proteomics and transcriptomics data. The left panel shows the regulation of genes and proteins in the IG group compared with the control group. The right panel shows the regulation of genes and proteins in the IV group compared with the control group. **(F)** KEGG pathway analysis revealed the top 20 pathways based on the same regulation of proteins and genes between the control and IV groups.

The proteomics and transcriptomics profiles from the liver samples in different groups (control, IV and IG) were evaluated using PCA and PLS-DA score plots. Proteomics and transcriptomics analyses yielded similar PCA and PLS-DA score plots. The IV group was located far from the control group, and the control group was not separated from the IG group in contrast to that observed in the PCA score plot (Figures 3A,C). These results indicated that intravenous injection with MSNs was significantly different from the control and IG groups. It was difficult to distinguish between the control group and IG group. PLS-DA presented good separation between the IV group, IG group and control group (Figures 3B,D).

A total of 8,190 proteins and 27,185 expressed genes were identified (Supplementary Figure S3). Upon oral administration of MSNs, 2 differentially expressed proteins and 42 differentially expressed genes were identified compared to the control group. Of these, 1 protein and 8 genes were upregulated, and 1 protein and 34 genes were downregulated. No overlap was noted between the regulated proteins and genes. Under intravenous injection with MSNs, 1,509 differentially expressed proteins and 1,376 differentially expressed genes were identified compared to the control group. Of these, 1,609 proteins and 1,250 genes were upregulated, and 781 proteins and 126 genes were downregulated. In total, 175 identical upregulations and 15 downregulations were noted between proteins and genes (Figure 3E, Supplementary Table S2). The 2 differentially expressed proteins in the IG group compared with the control group were Cyp2c39 and Plin2, whereas Cyp2c39 and Plin2 genes were not differentially regulated (Supplementary Table S3). KEGG pathway analysis was used to analyze the significant proteins and genes noted between the IV group and control group. Based on KEGG enrichment pathway analysis, we focused on metabolic pathways and inflammation (Figure 3F).

### 3.5 Energy Metabolism and Oxidative Phosphorylation

**FIGURE 4:**
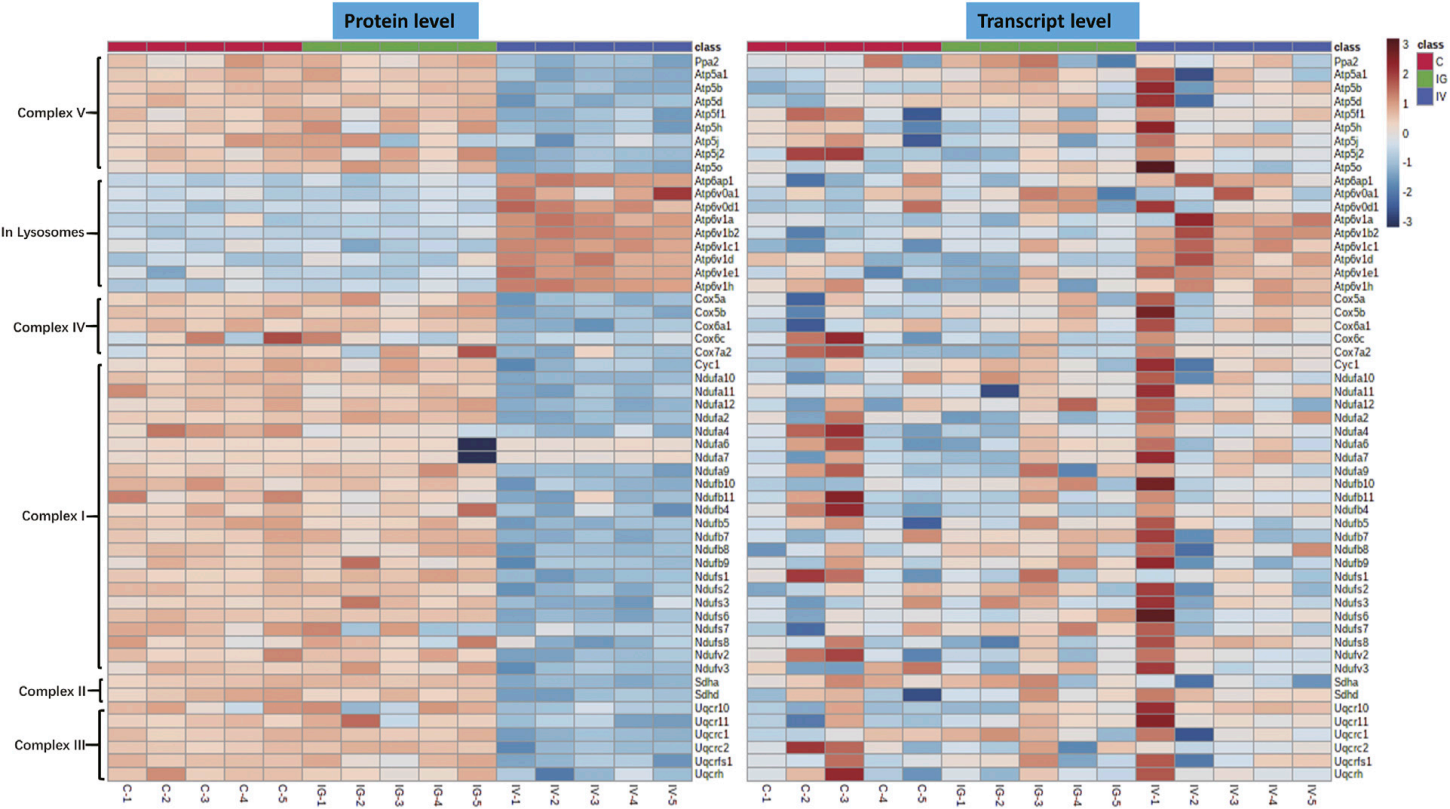
Effect of Mesoporous Silica Nanoparticles (MSNs) administered by different routes (IV and IG) on mitochondrial oxidative phosphorylation complexes. Heatmaps of genes and proteins involved in respiratory chain complexes I, II, III, IV and V of mitochondria and V-type proton ATPase of lysosomes.

The mitochondrion is a very important subcellular organelle given its role ROS and energy metabolism. Figure 4 shows that the levels of proteins of respiratory chain complexes I, II, III, IV and V of mitochondrion were obviously decreased and the levels of proteins of V-type proton ATPase of lysosomes were increased in the IV group compared with the control group. However, the IG group exhibited no changes in mitochondrial proteins. The mRNA expression levels of respiratory chain complexes were not significantly altered in the IV group and IG group compared with the control group.

**FIGURE 5:**
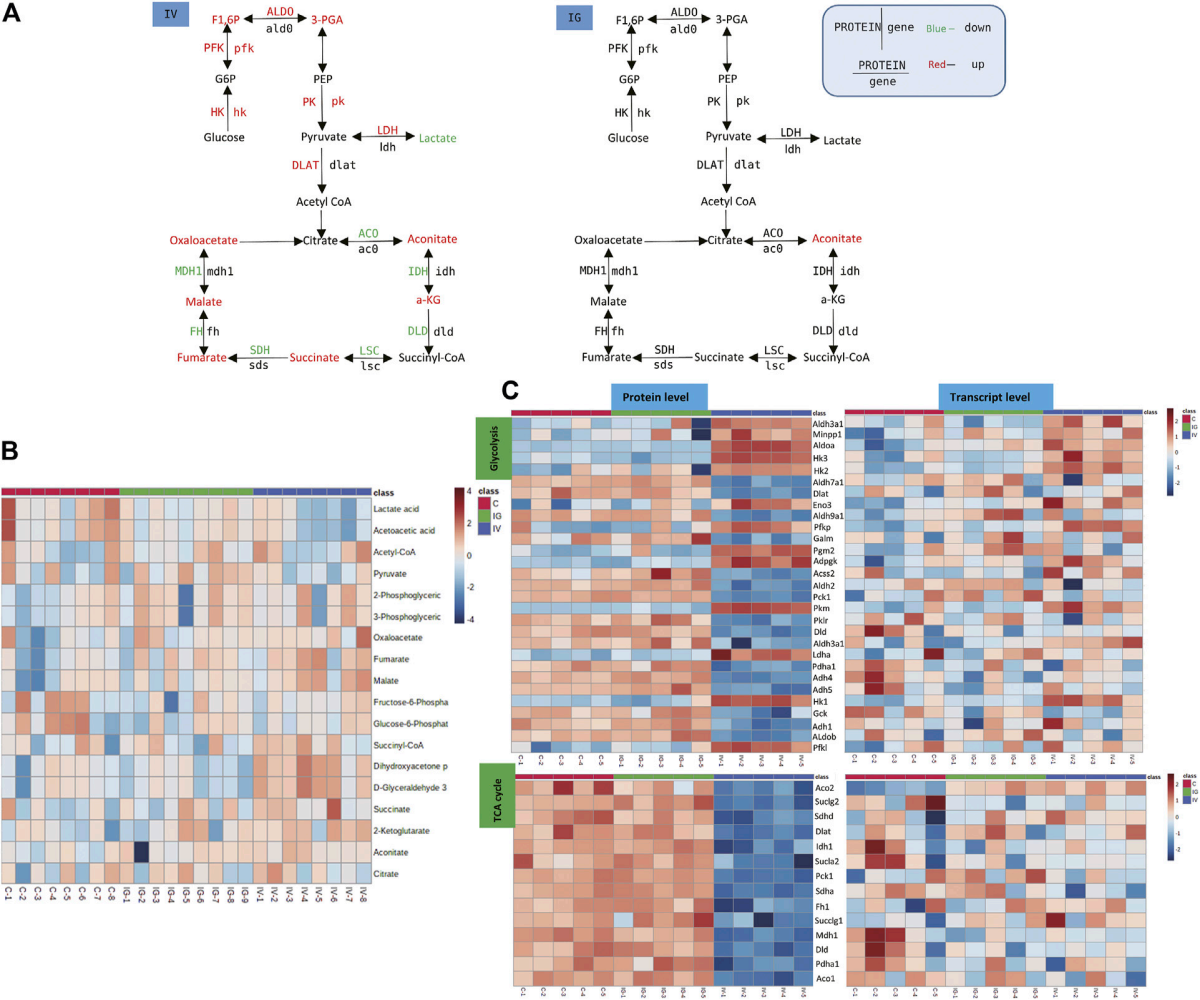
Effect of Mesoporous Silica Nanoparticles (MSNs) on the TCA cycle in mitochondria and glycolysis based on different routes of administration (IV and IG). **(A)** Schematic of glycolysis and the TCA cycle pathway. Red indicates significant upregulation, and green indicates significant downregulation. **(B)** Heatmap of metabolite concentrations of glycolysis and the TCA cycle pathway. **(C)** Heatmaps of glycolysis and the TCA cycle pathway based on proteomics and transcriptomics.

Lactate levels were significantly decreased in the IV group compared with the control group. In addition, the levels of 3-phosphoglyceric acid (3-PGA), fructose 1,6-bisphosphate (F1,6P), aconitate, alpha-ketoglutarate (a-KG), succinate, oxaloacetic acid, fumarate and malate were significantly increased in the IV group, and aconitate level was significantly increased in the IG group compared with the control group (Figure 5A, Supplementary Figure S1). The heatmaps in Figures 5B,D display the expression changes in the metabolite, protein and transcript levels of carbohydrates of the TCA cycle and glycolysis. These results showed significant differences between the IV group and the control group. Proteins and genes involved in energy metabolism, including hexokinase (HK), 6-phosphofructokinase 1 (PFK) and pyruvate kinase (PK), were upregulated (Figure 5A, Supplementary Figure S2). The data demonstrated that intravenous injection altered TCA cycle and glycolysis, whereas oral administration had minimal effects.

### 3.6 Antioxidant Pathway

**FIGURE 6:**
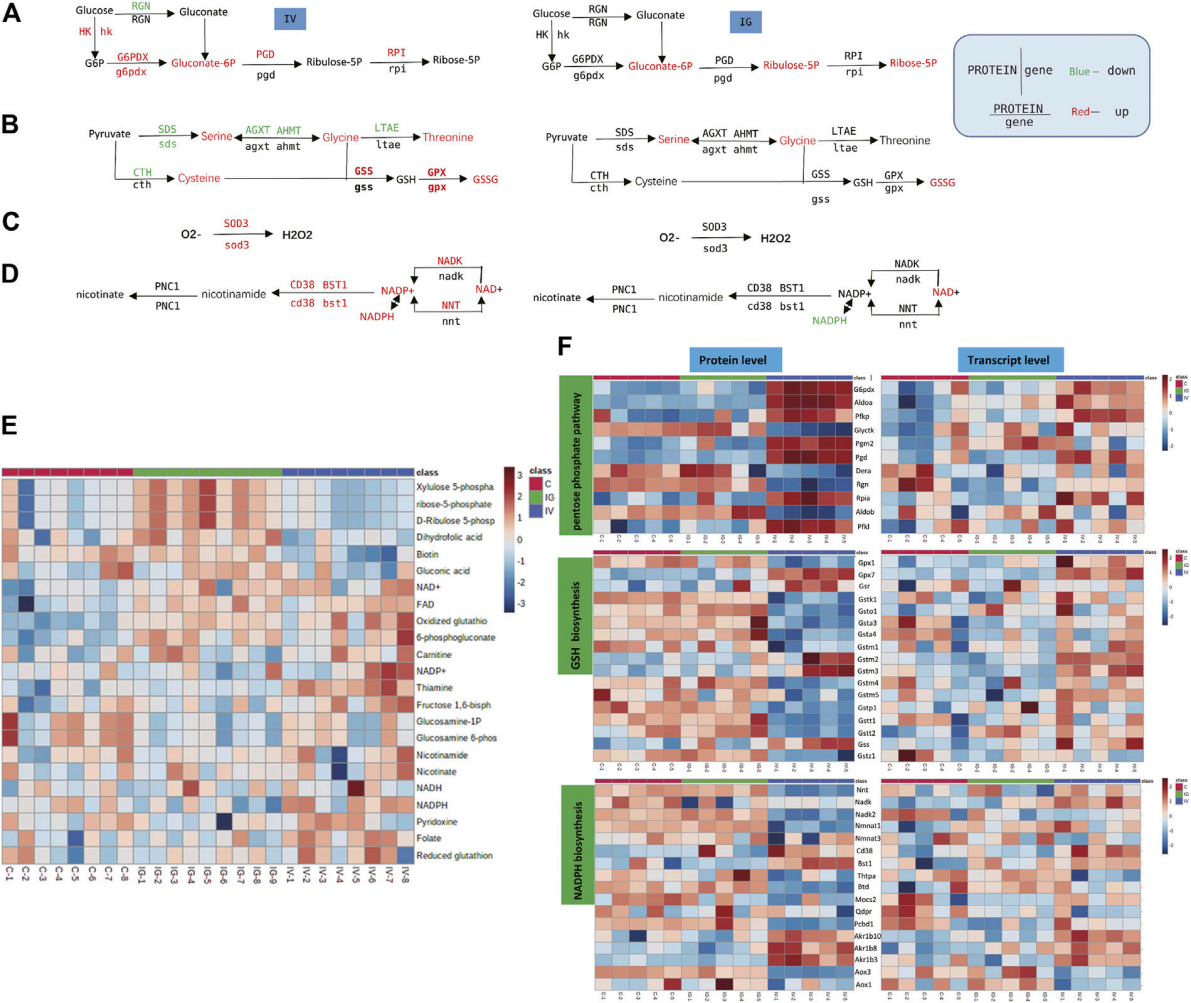
Effect of Mesoporous Silica Nanoparticles (MSNs) on the antioxidant pathway based on different routes of administration (IV and IG). **(A–D)** Schematic of PPP, glutathione biosynthesis, SOD3 antioxidation and NADPH biosynthesis. Red indicates significant upregulation, and green indicates significant downregulation. **(E)** Heatmap of metabolite concentrations of PPP, glutathione and NADPH biosynthesis. **(F)** Heatmaps of PPP, glutathione and NADPH biosynthesis based on proteomics and transcriptomics.

The antioxidant pathway in the mouse liver was significantly affected by oral administration or intravenous injection of MSNs. The PPP, glutathione, NADPH biosynthesis pathways and SOD3 reduce ROS to maintain redox equilibrium. We found significant upregulation of serine, glycine, 6-phosphogluconate, biotin, thiamine, FAD, NAD+, NADPH, NADP+ and oxidized glutathione and downregulation of folate in the IV group compared with the control group. The levels of serine, glycine, 6-phosphogluconate, ribose 5-phosphate, dihydrofolic acid, FAD, NAD+ and oxidized glutathione were significantly increased, and NADPH and GSH/GSSG levels were downregulated in the IG group compared with the control group (Figures 6A–D, Supplementary Table S1). The heatmaps in Figures 6E,F display the expression changes in the metabolite, protein and transcript levels associated with PPP, glutathione and NADPH metabolism. These results revealed significant differences between the IV group and the control group. The upregulated glutathione and NADPH metabolism proteins and genes included CD38, glutathione peroxidase (GPX) and glutathione S-transferase (GST) (Figures 6A–D, Supplementary Table S2).

### 3.7 Purine and Pyrimidine Metabolism and Biosynthesis

**FIGURE 7:**
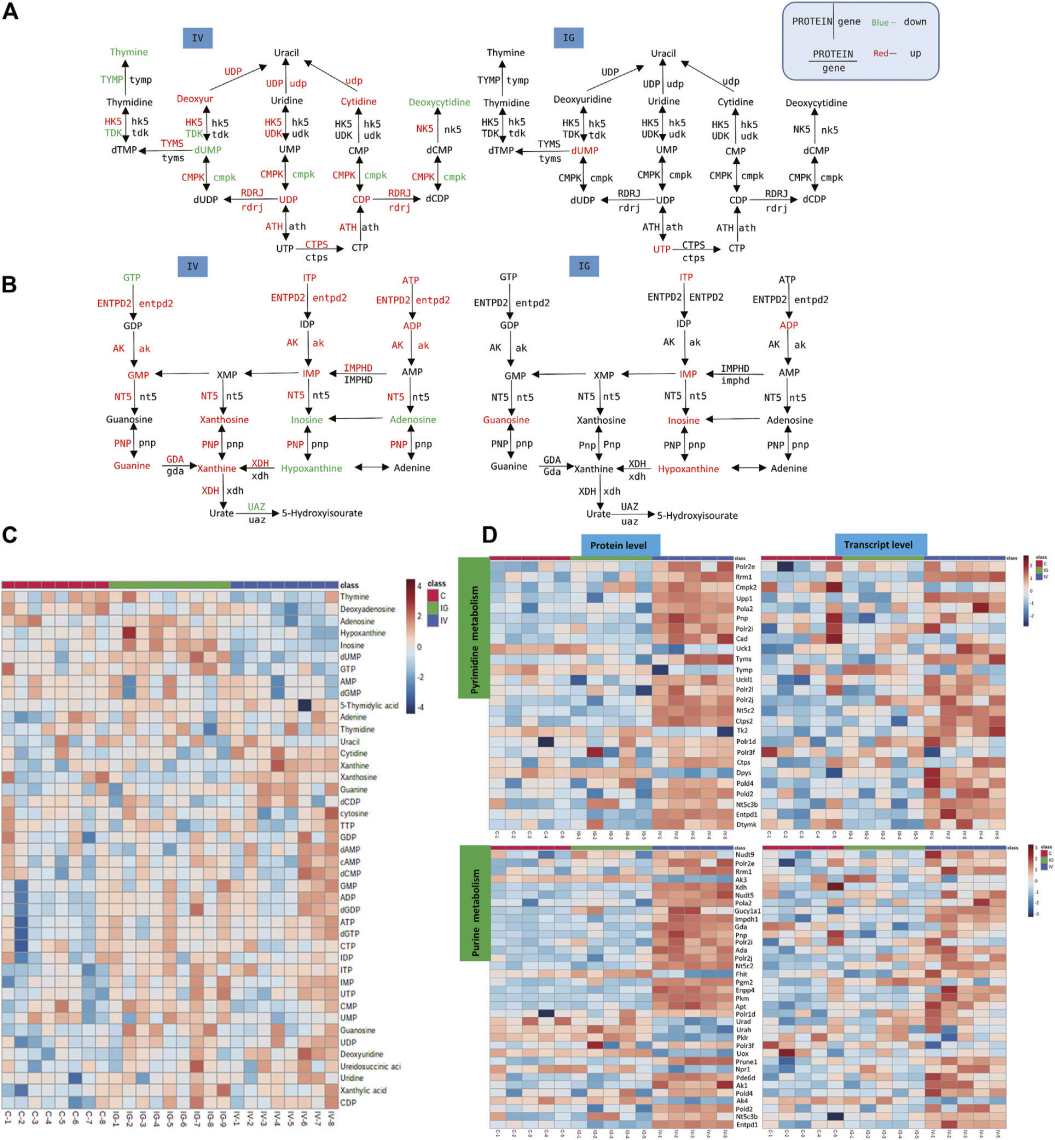
Effect of Mesoporous Silica Nanoparticles (MSNs) on purine and pyrimidine nucleotides based on different routes of administration (IV and IG). **(A, B)** Schematic of purine and pyrimidine nucleotide metabolism; red indicates significant upregulation, and green indicates significant downregulation. **(C)** Heatmap of concentrations of metabolites of purine and pyrimidine nucleotide metabolism. **(D)** Heatmaps of purine and pyrimidine nucleotide metabolism based on proteomics and transcriptomics.

A number of purine and pyrimidine nucleotides were aberrantly altered in mice treated with MSNs orally administered or intravenously injected via the tail vein. For instance, compared with the control group, the levels of GMP, IMP, ITP, UDP, ATP, ADP, dGTP, dGDP, guanine, xanthine, xanthosine, cytidine and deoxyuridine were notably increased, and the levels of dUMP, hypoxanthine, thymine, deoxyadenosine, GTP, inosine and adenosine were significantly reduced in the IV group. However, the levels of IMP, ITP, UTP, ADP, dUMP, guanosine, inosine and hypoxanthine were significantly increased in the IG group compared to the control group (Figure 7D, Supplementary Table S1). The heatmaps in Figures 7C,E display the expression changes in the metabolite, protein and transcript levels of nucleotides and nucleosides. These results revealed significant differences between the IV group and the control group. Numerous proteins and genes associated with purine and pyrimidine nucleotides were upregulated: 3′,5′-cyclic-nucleotide phosphodiesterase (PDE), adenylate kinase (AK), ribonucleoside-diphosphate reductase subunit M2 (RRM2), adenosine triphosphatase (ENTDP2) and uridine phosphorylase (UPP) (Figures 7A,B).

## 4 Discussion

Our data showed that intravenous injection of MSNs caused significant changes in parameters related to liver function, histopathological sections, and biological system results based on metabolomics, genomics, and proteomics. We observed obvious injury to the liver, upregulation of inflammatory factors of the liver and induced glycolysis, the tricarboxylic acid cycle, and oxidative phosphorylation related to mitochondrial energy metabolism, enhanced succinic acid and glutathione synthesis associated with inflammation and oxidative stress, and an abnormal PPP activity for nucleic acid synthesis. Oral administration of MSNs also increased levels of inflammatory factors and altered metabolomics, transcriptomics and proteomics profiles. However, the effect was significantly weaker than that of intravenous administration, even though the dose was much higher than that of intravenous injection. After intravenous injection, all the particles directly enter the systemic circulation and are more likely to accumulate in the liver and thus cause liver damage (Cho et al., 2009; Geraets et al., 2014; Elgrabli et al., 2015). However, after oral administration, most of the particles gather in the gut, and only a small amount of these particles enter the liver via the hepatic portal vein and then systemic circulation. We presume it is the key reason why liver function was affected by oral particles, but the effect was weaker than that of intravenous injection. In support of this hypothesis, a previous study showed that liver tissue is highly exposed to these refractory particles after injection, and liver tissue is also exposed to these particles after oral administration (Cho et al., 2009; Geraets et al., 2014; Elgrabli et al., 2015). Consistently, a number of studies (Yu et al., 2012; Dogra et al., 2018) have reported that the particle exposure level in liver tissue increased significantly after oral administration of MSNs, suggesting that oral nanoparticles can be absorbed into the liver through the intestine and ultimately affect liver tissue.

Based on systematic biological proteomics, transcriptome and metabolomics data, the effects of intravenous and gavage administration on liver tissues were evaluated. We observed that MSN administration, especially intravenous administration, significantly affects the metabolism of small molecules, proteins and genes in the liver. Transcriptomics and proteomics revealed a mutual and consistent effect of MSNs on GPX, SOD3 and G6PD genes/proteins related to oxidative stress; HK and PFK genes/proteins related to glycolysis; and UUP, AK and RRM2 genes/proteins related to nucleic acid synthesis. These results suggest that liver injury is closely related to the nucleic acid synthesis pathway, oxidative stress, glycolysis and mitochondrial energy metabolism. The metabolomics results showed significant changes in many metabolites involved in metabolism, and these results are consistent with the results of transcriptomics and proteomics studies. For example, the perturbed metabolites GSSG, NADPH, NADP+, succinic acid and hypoxanthine are closely related to oxidative stress as uncovered by transcriptomic and proteomic data. IMP, UDP, and GMP are key metabolites involved in nucleic acid synthesis. In addition, the TCA intermediates, F1,6P and 3-PGA are important in glycolysis/mitochondrial energy metabolism. The integrated analysis of proteomic, transcriptomic and metabolomics data suggested consistent effects of MSNs on key metabolic pathways, especially the nucleic acid synthesis pathway, oxidative stress, and glycolysis/mitochondrial energy metabolism in the liver.

To our surprise, transcriptions and proteins analysis indicated inconsistent data, i.e., the expression at protein level showed regularly consistent within each group, and distinctly different from the other groups, while the expression at transcription level did not. Usually, the expression at transcription and protein levels matches well with each other, and with the activities, for examples, the mRNA and protein expression levels of isocitrate dehydrogenase, succinate dehydrogenase, malate dehydrogenase and other enzymes in the respiratory chain complex protein and TCA cycle are consistent. However, occasionally, the correlations don’t match between mRNA expression level and protein level in microorganism and mammalian (Gygi et al., 1999; Maier et al., 2009; Rossignol et al., 2009; Schwanhüusser et al., 2011; Liu et al., 2016). Although the underlying mechanism is not well understood, data suggested that ROS and/or oxidative stress was involved in their diverse effect on mRNA and protein expression levels. For an example, Song et al. (2022) reported that, due to the time lag effect in protein modification, gene transcription and translation, the expressions of SOD and GPX subtype genes did not change in accordance with those at protein levels. It was presumed that the oxidative stress or altered ROS level perturbed mRNA translation efficiency, and also affected protein degradation rates and the folding and modification efficiency of proteins, e.g., in mitochondria and nucleus (Tan et al., 2017; Latonen et al., 2018; Sun et al., 2019). In this study, we observed a significant oxidative stress in liver, indicating that the inconsistency of expression at mRNA and protein levels was involved in oxidative stress induced by MSNs.

Mitochondria are the primary site of cellular energy production, and the TCA cycle is the main pathway responsible for energy generation. The liver is not only the material transformation center of the body but also a center with intensive energy loading (Akram, 2014; Panieri and Santoro, 2016). Our data showed that after MSN injection, mitochondrial respiratory chain protein complexes I, II, III, IV and V decreased. In addition, oxidative phosphorylation was reduced, and circulating TCA intermediates generally increased. These results suggest that TCA metabolism was affected and ATP production decreased. To compensate for the loss caused by reduced energy production, cells generally tend to upregulate glycolysis. Therefore, the F1,6P and 3PGA metabolites as well as HK, PFK, PK genes and proteins in the glycolysis pathway were upregulated. On the other hand, nanoparticles typically induce oxidative stress and mitochondrial dysfunction (Li et al., 2016; Lim et al., 2019; Sun et al., 2019). To alleviate mitochondrial damage, the downregulation of the mitochondrial respiratory chain was observed and characterized by reduced mitochondrial respiratory chain protein complexes I, II, III, IV and V, including TCA metabolism. Because the production of ROS greatly contributes to oxidative stress and inflammatory factors (Hur and Gray, 2011; Nishijima et al., 2017; Snezhkina et al., 2020), the elevation of inflammatory factors indicates that MSN-induced inflammation occurs independently of ROS levels. Previous studies have shown that the accumulation of succinic acid, an intermediate substance in the TCA cycle, can stimulate inflammation and induce upregulation of inflammatory factors (Tannahill et al., 2013). Metabolomics data showed that MSNs injection led to a significant increase in succinic acid, indicating that it is related to inflammation and inflammatory factors (Lim et al., 2019; Mohamed et al., 2020). Moreover, liver injury triggers the repair and regeneration of liver cells. Therefore, metabolic pathway analysis showed an enhanced PPP pathway in the liver, which is primarily responsible for increased synthesis of nucleic acids for the construction of genetic materials. Compared with intravenous injection, intragastric administration of MSNs did not induce abnormalities in mitochondrial function; however, distinct changes in antioxidant pathways, such as glutathione synthesis and PPP, and the key metabolites of hypoxanthine and NADPH synthesis were noted. In general, the milder effects of oral administration on metabolism and metabolic pathways can be attributed to the reduced amount of particles entering the liver compared with that observed with intravenous injection.

The literature (Lu et al., 2010; Yu et al., 2012; Fu et al., 2013; Isoda et al., 2013; Hassankhani et al., 2015; Chatterjee et al., 2016; Abdelhalim et al., 2018; Li et al., 2018; Abdel-Latif et al., 2021; Sun et al., 2021) and our previous studies (Lu et al., 2010; Yu et al., 2012; Fu et al., 2013; Isoda et al., 2013; Hassankhani et al., 2015; Chatterjee et al., 2016; Abdelhalim et al., 2018; Li et al., 2018; Abdel-Latif et al., 2021; Sun et al., 2021) have shown that silica particles with particle sizes of 10–1000 nm cause significant damage to the liver, whereas particles with sizes greater than 100 nm cause significant damage to the kidney. To study the effect of particles on the liver, we selected MSNs with an average particle size of 80 nm with a normal particle size distribution. Our data showed that MSNs have a significant impact on liver tissue morphology, cell function and metabolism. This study focuses on nondegradable particles, so their effects on the function and metabolism of the liver cannot be directly extrapolated to degradable particles. Considering that undegradable and degradable particles have different dynamic fates *in vivo*, the influence of degradable particles on tissues and organs, especially liver tissue, needs to be further studied and clarified.

## 5 Conclusion

Intravenous injection of MSNs induced inflammation, and significant liver toxicity was noted. Based on metabolomics, proteomics and transcriptomics analyses, this systematic biological study suggested perturbed mitochondrial energy metabolism of the TCA, oxidative phosphorylation and glycolysis and stimulated oxidative stress involved in the synthesis of the glutathione pathway and nucleotides via the PPP. Oral administration of MSNs did not induce distinct hepatic injury but did stimulate inflammatory factors and affected metabolic pathways involved in the PPP, glutathione synthesis and oxidative stress albeit to a lesser extent than intravenous injection, even at much higher doses. The data suggested that intravenous injection of nanoparticles of pharmaceutical excipients substantially affected hepatic function and metabolism and induced oxidative stress in the liver.

## Data Availability

The datasets presented in this study can be found in online repositories. The names of the repository/repositories and accession number(s) can be found below: mass spectrometry proteomics data have been deposited to the ProteomeXchange Consortium via the PRIDE partner repository with the dataset identifier PXD030757 and transcriptomics data can be found here: National Center for Biotechnology Information (NCBI) BioProject database under accession numbers PRJNA794322.
