# Supplementary material for: Integrative Metabolomics, Proteomics and Transcriptomics Analysis Reveals Liver Toxicity of Mesoporous Silica Nanoparticles
Source: Front Pharmacol. 2022 Jan 27;13:835359. doi: 10.3389/fphar.2022.835359 (PMC8829009; doi:10.3389/fphar.2022.835359)
Supplement: Supplementary file 1 [file DataSheet1.docx]

Supplementary Material

# Method

**GC-MS parameters**

Shimadzu GCMSQP2010 (Shimadzu Corp., Tokyo, Japan) equipped with a RTx-5MS column (30 mm × 0.25 mm i.d. fusedsilica capillary column chemically bonded with a 0.25-μm cross bond, 5% diphenyl/95% dimethyl polysiloxane, Restek Corporation, PA, USA). The molecules were ionized with a current beam of 70 eV. Mass spectra were acquired from m/z 50 to 680 with a detector voltage of -1050 V. The column temperature was initially kept at 80°C for 3 min and then was increased to 300°C for another 5 min.

**LC-MS parameters**

The HPLC separation was carried out on a Waters Amide XBridge HPLC column (4.6×100 mm, 3.5 μm, Waters, Milford, MA, USA). The chromatographic column was maintained at 30°C. Mobile phase consisted of mobile phase A and mobile phase B (acetonitrile). Phase A was an aqueous solution containing 5 mM ammonium acetate. The pH value was adjusted to 9 with ammonia water, subsequently acetonitrile was added to mobile phase A to 5%. Gradient elution is used for chromatographic separation, and the gradient procedures are set as follows: 0-3 min 85% B, 3-6 min 85%-30% B, 6-15 min 30%-2% B, 15-18 min 2% B, 18-19 min 2%-85% B, 19-26 min, 85% B. And the flow rate was 0.4 mL/min. The mass spectrometer was detected in negative electrospray ionization (ESI) mode. The ion mode scanning scanning range and sub ion scanning range was 50-1000 and 50-900 m/z, respectively. Ion Source Gas 1 (GAS 1) ion SourceGas 2 (GAS 2) and air curtain gas (Curtain Gas) was 33, 33 and 25 psi, respectively. Other MS parameters were as follows: Ion spray voltage: -4500 V; Turbo spray temperature: 500 C; Declustering Potential (DP) voltage: 93 V; TOF MS scan fragmentation voltage (Collision Energy, CE): -10 V; The accurate mass numbers by Calibration Delivery System (CDS) correction. Each of the 6 samples is corrected automatically with a correction mass.

Table S1. Significantly altered metabolites in mice liver treated with MSNs.

|  | metabolites | control-iv | | control-ig | |
| --- | --- | --- | --- | --- | --- |
| category |  | p-value | foldchange | p-value | foldchange |
| nucleotides, nucleosides | dUMP | 0.027333 | 0.71875 | 0.003324 | 1.688028 |
|  | GMP | 0.046007 | 1.235283 | 0.185824 | 1.098798 |
|  | IMP | 0.022349 | 1.458175 | 0.021079 | 1.425328 |
|  | Xanthylic acid | 0.103037 | 1.232522 | 0.00876 | 1.331721 |
|  | Guanine | 0.036066 | 1.397851 | 0.46836 | 1.065465 |
|  | Hypoxanthine | 0.031549 | 0.804483 | 0.045709 | 1.461198 |
|  | Thymine | 0.039994 | 0.720591 | 0.571028 | 0.945293 |
|  | Xanthine | 0.009325 | 1.268629 | 0.786097 | 0.985028 |
|  | Xanthosine | 0.018253 | 1.66335 | 0.510872 | 0.854251 |
|  | Cytidine | 0.031743 | 1.4979 | 0.474568 | 0.925023 |
|  | Deoxyadenosine | 0.012021 | 0.748353 | 0.097426 | 0.846493 |
|  | Deoxyuridine | 0.007351 | 1.880233 | 0.122569 | 1.390215 |
|  | Inosine | 0.820658 | 0.973813 | 0.010449 | 1.402418 |
|  | ATP | 0.031508 | 1.490655 | 0.070792 | 1.342135 |
|  | ADP | 0.015031 | 1.319683 | 0.005976 | 1.278801 |
|  | dGTP | 0.031508 | 1.490655 | 0.070792 | 1.342135 |
|  | dGDP | 0.017536 | 1.331091 | 0.010266 | 1.338642 |
|  | GTP | 0.049792 | 0.724955 | 0.699986 | 1.066107 |
|  | ITP | 0.00079 | 1.483425 | 0.001108 | 1.526667 |
|  | UTP | 0.085047 | 1.689807 | 0.043389 | 1.74849 |
|  | UDP | 0.028381 | 1.263602 | 0.050063 | 1.213694 |
|  | Adenosine | 0.037553 | 0.786189 | 0.604981 | 1.050212 |
|  | Guanosine | 0.139933 | 1.32648 | 0.009829 | 1.639923 |
| Glycolysis, gluconeogenesis, pentose phosphate pathway, TCA cycle | Fructose 1,6-P | 0.038368 | 1.597694 | 0.923105 | 0.948829 |
|  | Oxaloacetic acid | 0.016878 | 1.344318 | 0.065974 | 1.224534 |
|  | phenylpyruvate | 0.023646 | 1.19918 | 0.04218 | 1.149555 |
|  | Glyceraldehyde 3-P | 0.001961 | 2.076315 | 0.285066 | 0.86024 |
|  | Lactate acid | 0.022304 | 0.779255 | 0.09948 | 0.874125 |
|  | Aconitate | 0.022438 | 1.362385 | 0.030269 | 1.263861 |
|  | Fumarate | 0.026462 | 1.207418 | 0.123375 | 1.118495 |
|  | Malate | 0.040853 | 1.258928 | 0.450182 | 1.073689 |
|  | Succinate | 0.018961 | 1.433603 | 0.152101 | 1.209386 |
|  | 2-Ketoglutarate | 0.014262 | 1.229034 | 0.426027 | 1.078144 |
|  | 6-phosphogluconate | 0.028192 | 1.879683 | 0.000227 | 1.934429 |
|  | 2-Dehydro -gluconate | 0.382987 | 1.127031 | 0.001725 | 1.515404 |
|  | ribose-5-phosphate | 0.458956 | 0.953434 | 0.000879 | 1.319298 |
|  | Ribulose 5-phosphate | 0.458956 | 0.953434 | 0.000879 | 1.319298 |
|  | Xylulose 5-phosphate | 0.458956 | 0.953434 | 0.000879 | 1.319298 |
|  | Glucosamine-1-P | 0.119997 | 0.786849 | 0.004784 | 0.654605 |
|  | Glucosamine 6-P | 0.119997 | 0.786849 | 0.004784 | 0.654605 |
|  | Glycerol-3-P | 0.292697 | 1.259988 | 0.033862 | 1.512472 |
| Amino acid | citrulline | 0.00053 | 1.43443 | 0.333487 | 1.058291 |
|  | Ornithine | 0.024915 | 1.550106 | 0.577425 | 1.134035 |
|  | Asparagine | 0.01551 | 1.361039 | 0.888381 | 0.939059 |
|  | glutamate | 0.142047 | 1.260963 | 0.025438 | 1.684728 |
|  | glycine | 0.000136 | 1.249345 | 0.00202 | 1.165731 |
|  | histidine | 0.000111 | 1.258659 | 1.44E-05 | 1.251664 |
|  | isoleucine | 0.007988 | 1.253582 | 0.295913 | 1.070269 |
|  | lysine | 0.004132 | 1.313253 | 0.764195 | 1.026413 |
|  | methionine | 0.00037 | 1.410224 | 0.001969 | 1.33281 |
|  | Phenylalanine | 0.01186 | 1.257229 | 0.020808 | 1.137064 |
|  | proline | 0.001702 | 1.424968 | 0.093924 | 1.112734 |
|  | serine | 0.000358 | 1.410699 | 0.012751 | 1.260664 |
|  | tryptophan | 0.000511 | 1.256398 | 0.921508 | 1.037926 |
|  | valine | 0.003661 | 1.269356 | 0.128231 | 1.091196 |
|  | Leucine | 0.004819 | 1.212062 | 0.190266 | 1.080501 |
|  | Isoleucine | 0.032584 | 1.167271 | 0.855316 | 1.013269 |
|  | Threonine | 0.012668 | 1.186925 | 0.065937 | 1.124343 |
|  | b-Alanine | 0.012094 | 1.285915 | 0.431489 | 0.933962 |
|  | Aspartate | 0.002313 | 1.468247 | 0.011678 | 1.398668 |
|  | Cysteine | 0.004647 | 1.579387 | 0.344246 | 1.101852 |
|  | Glutamate | 0.214003 | 1.204556 | 0.026651 | 1.55219 |
| Fatty acid, Ketone body | Cis-9-Hexadecenoic Acid | 0.001761 | 0.714023 | 0.622998 | 1.050166 |
|  | Undecanedioic acid | 0.036098 | 0.574009 | 0.622923 | 0.904926 |
|  | Acetoacetic acid | 0.018165 | 0.777593 | 0.126374 | 0.889526 |
|  | 1-Monolinolein | 0.000587 | 0.802993 | 0.008463 | 1.178419 |
| Vitamine, GSH, NADPH | Biotin | 0.001797 | 0.498878 | 0.504261 | 0.954081 |
|  | Folate | 0.013212 | 1.358429 | 0.943848 | 1.022898 |
|  | Dihydrofolic acid | 0.364612 | 0.792895 | 0.029583 | 1.596953 |
|  | Thiamine | 0.000103 | 1.422248 | 0.356901 | 1.060306 |
|  | FAD | 0.002011 | 1.276451 | 0.000357 | 1.305351 |
|  | NAD+ | 0.01679 | 1.192463 | 0.034527 | 1.150762 |
|  | NADPH | 0.024913 | 1.423293 | 0.011764 | 0.610046 |
|  | NADP+ | 0.046002 | 1.528132 | 0.338506 | 0.898290 |
|  | Oxidized glutathione | 0.00192 | 1.481228 | 3.66E-06 | 1.391327 |
| Others | methylcysteine | 0.020741 | 1.40005 | 0.068706 | 1.454663 |
|  | Creatinine | 0.001221 | 1.689087 | 0.155034 | 1.216994 |
|  | Butanoic acid | 0.014711 | 1.702882 | 0.063915 | 1.309439 |
|  | Acetamide | 0.0054 | 1.286721 | 0.138472 | 1.137989 |
|  | Cystathionine | 0.03442 | 0.702861 | 0.291335 | 1.202855 |
|  | Ribitol | 0.006444 | 0.73933 | 0.174831 | 1.128193 |
|  | Myo-Inositol | 9.28E-06 | 1.53476 | 0.360342 | 1.054467 |
|  | Myo-Inositol-2-P | 0.029245 | 0.834636 | 0.425515 | 0.954421 |
|  | Cholesterol | 0.01092 | 1.097128 | 0.15266 | 1.039539 |

Table S2. The same regulation of protein and gene in mice liver in response to MSNs exposure by different exposure ways (IV and IG) using proteomics and transcriptomics.

|  | up-regulation | down-regulation |
| --- | --- | --- |
| Control-IG | / | / |
| Control-IV | 6-Sep, AB124611, Aif1, Ak1, Anxa1, Anxa3, Anxa5, Ap1s2, Apbb1ip, Arhgap25, Arhgap4, Arhgap45, Arhgdib, Arhgef6, B4galnt1, Basp1, Bin2, Camp, Capg, Cbr3, Cd14, Cd200, Cd300a, Cd44, Cd48, Cd55, Cd63, Cd68, Cdk1, Chil3, Ckb, Cmtm3, Cnn2, Col3a1, Col5a2, Coro1a, Crip1, Csf1r, Cyba, Cybb, Dock10, Dock2, Dok1, Dok2, Ehbp1l1, Epb41l3, Fabp4, Fabp5, Fam49a, Fbln5, Fcer1g, Fcgr3, Fermt3, Fgr, Fhl1, Fhod1, Fscn1, Fxyd5, Fyb, G6pdx, Gbp2, Gbp8, Gimap3, Glipr2, Gm14548, Gng2, Gp1ba, Gpx7, Gramd1b, Gstm3, Gzma, H2-Ab1, H2-DMb2, H2-Q7, Hcls1, Hk1, Hk2, Hk3, Hmgb2, Hmox1, Ighg2b, Igkc, Igkv12-38, Iglc1, Inpp5d, Iqgap1, Itga2b, Itga6, Itgal, Itgam, Itgb2, Itgb3, Jchain, Kcnab2, Lbh, Lcp2, Lgals1, Lgals3, Lpcat1, Lpcat2, Lpl, Lpxn, Lrmp, Lrrc25, Ltf, Ly9, Lyz2, Mad2l1, Marcks, Me2, Mmrn1, Mpo, Mrc2, Mvd, Myo1f, Myof, Ncf2, Nckap1l, Nfam1, Ngp, Nos2, Nsdhl, Oxct1, P2rx7, Parvb, Pdlim2, Pfkp, Pip4k2a, Pkm, Pla2g7, Pld4, Plek, Plekha2, Ppic, Ppm1h, Ppp1r18, Prex1, Ptprc, Pycard, Rab27b, Rab31, Rac2, Rap2b, Rassf2, Rbp1, Renbp, Rftn1, Rgs10, Ripk3, Serpinh1, Sh3bgrl3, Sh3bp1, Sh3kbp1, Sh3pxd2b, Sirpa, Slc11a1, Slc43a2, Smpdl3b, Sod3, Src, Stmn1, Susd2, Syk, Tagln2, Tbxas1, Thy1, Timd4, Tm6sf1, Tnc, Tnfaip8, Tnfaip8l2, Trpv2, Upp1, Vav1, Vcam1, Vim, Vnn3 | Acsm2, Arhgef26, Ces1f, Cyp2u1, Dhx58, Gck, Glo1, Hsd3b5, Mup16, Mup18, Mup19, Mup3, Mup9, Slc22a28, Ugt2b1 |

Table S3. The regulation of protein and gene between Control group and IG group in mice liver in response to MSNs exposure.

| Control-IG | Proteomics | Transcripromics |
| --- | --- | --- |
| up-regulation | Cyp2c39 | Nr1d1, Gm45044, B930025P03Rik, Muc16, Gm16001, Msln, Dbp, Lrp2 |
| down-regulation | Plin2 | Uhrf1, Derl3, C4a, Wsb1, Frk, Srebf1, Fos, Fam83f, Ly6c2, Sdf2l1, Slc10a2, Clic5, Npas2, Slc25a25, Il1b, Il1a, Olfm3, Herc6, Kbtbd8,, C330027C09Rik, Slc41a2, H2-Aa, Gbp7, Samd9l, Trim30d, H2-Eb1, BC023105, Tnfaip8l3, Gm15348, Ifit3, Ly6a, Tgtp2, Phf11b, Gbp6 |

Table S4. Primers used for gene expression analysis

| **Primers** | **Sequence (5'-3')** | **Strain** |
| --- | --- | --- |
| *TNF-a* | Forward CAGCCGATGGGTTGTACCTT | Mouse |
|  | Reverse ATAGCAAATCGGCTGACGGT |  |
| *IL-6* | Forward AGCCAGAGTCCTTCAGAGAGAT | Mouse |
|  | Reverse AGGAGAGCATTGGAAATTGGGG |  |
| *IL-1b* | Forward GTGTCTTTCCCGTGGACCTT | Mouse |
|  | Reverse AATGGGAACGTCACACACCA |  |
| β-Actin | Forward AACACCCCAGCCATGTACG | Mouse |
|  | Reverse ATGTCACGCACGATTTCCC |  |
| *GAPDH* | Forward TCACCAGGGCTGCTTTTAACT | Mouse |
|  | Reverse TTCCCGTTCTCAGCCTTGAC |  |


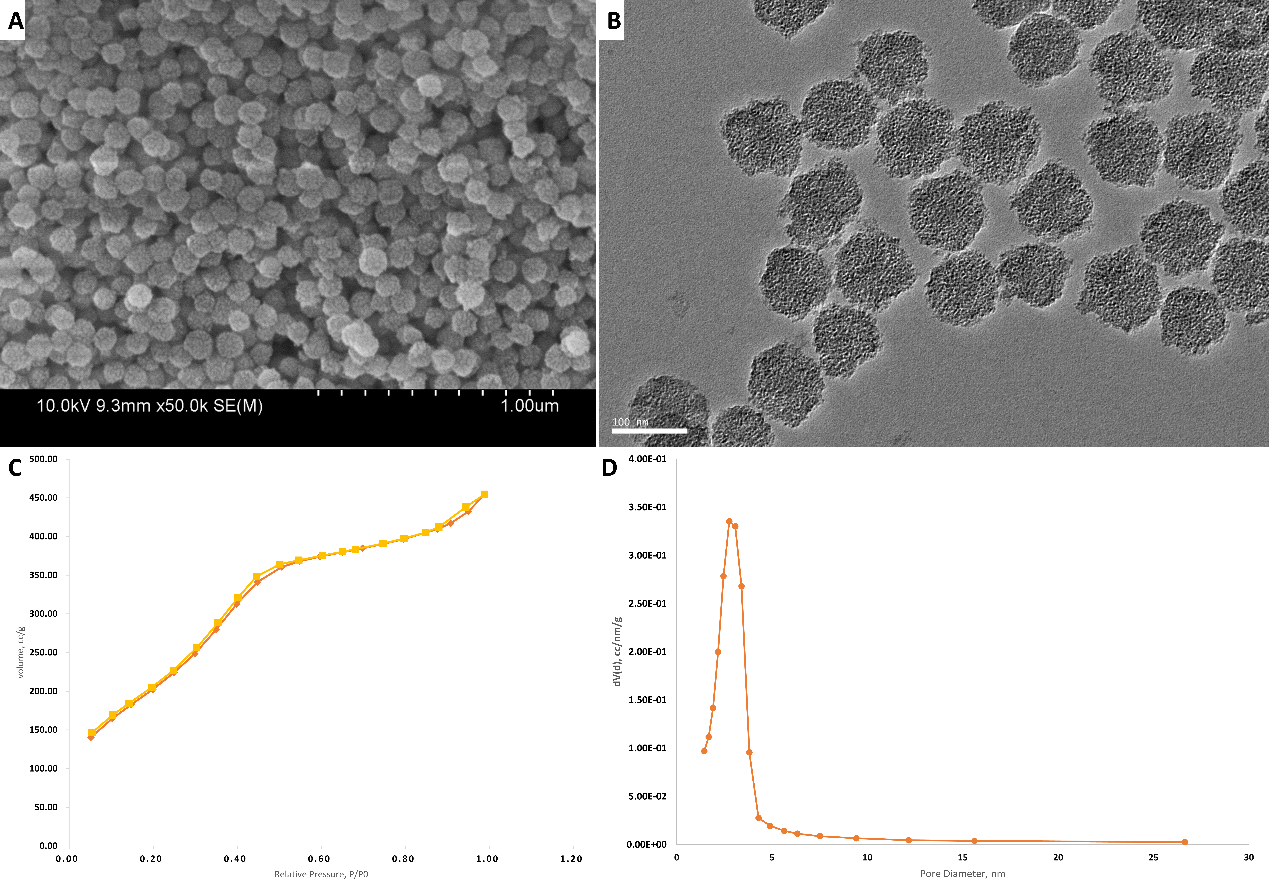


Figure S1. Characterization of MSNs(A) SEM image of MSNs, (B) TEM image of MSNs, (C) Nitrogen adsorption-desorption isotherms and (D) BJH pore-size distribution curves of MSNs.


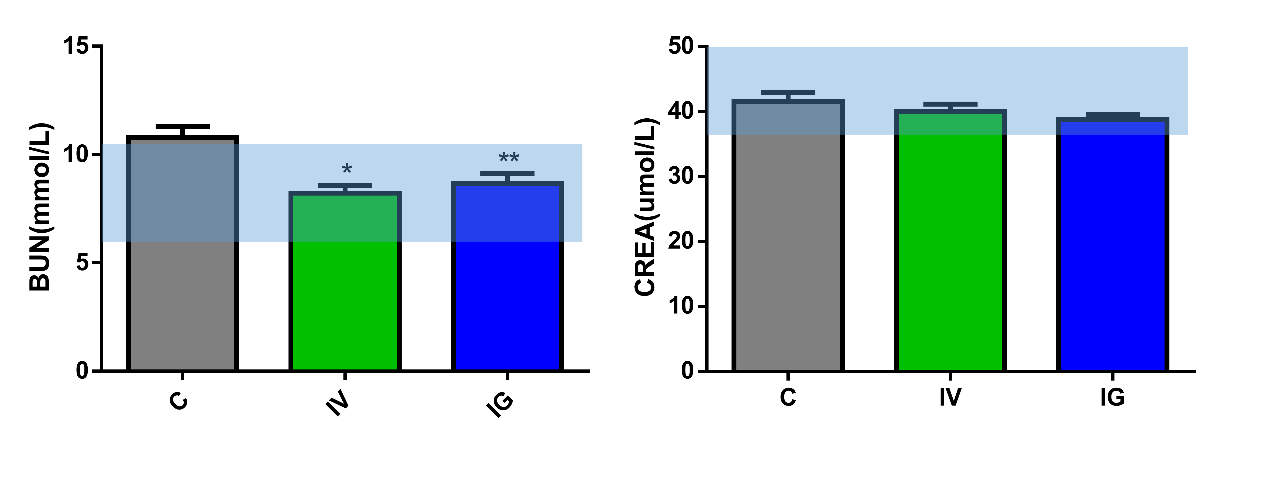


Figure S2. Effect of different exposure ways (IV and IG) on serum biochemistry BUN and CREA. ∗p < 0.05, ∗∗p < 0.01 compared with control. Light blue bars indicated the range of values obtained from healthy ICR mice.


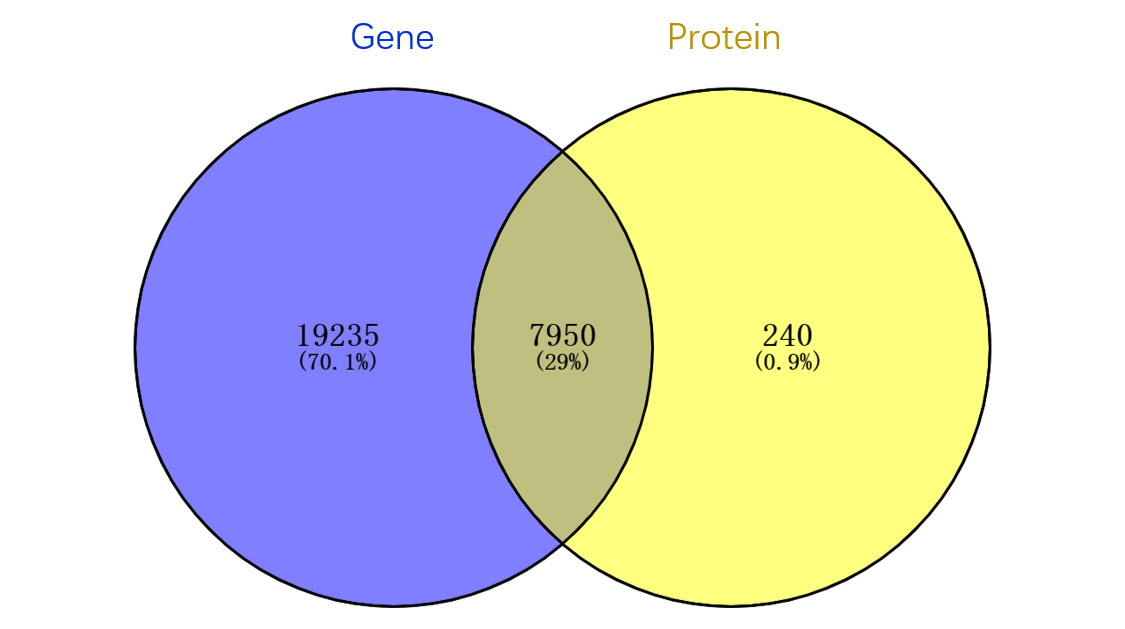


Figure S3. Venn diagram of the total correlation numbers between proteomics and transcriptomics.


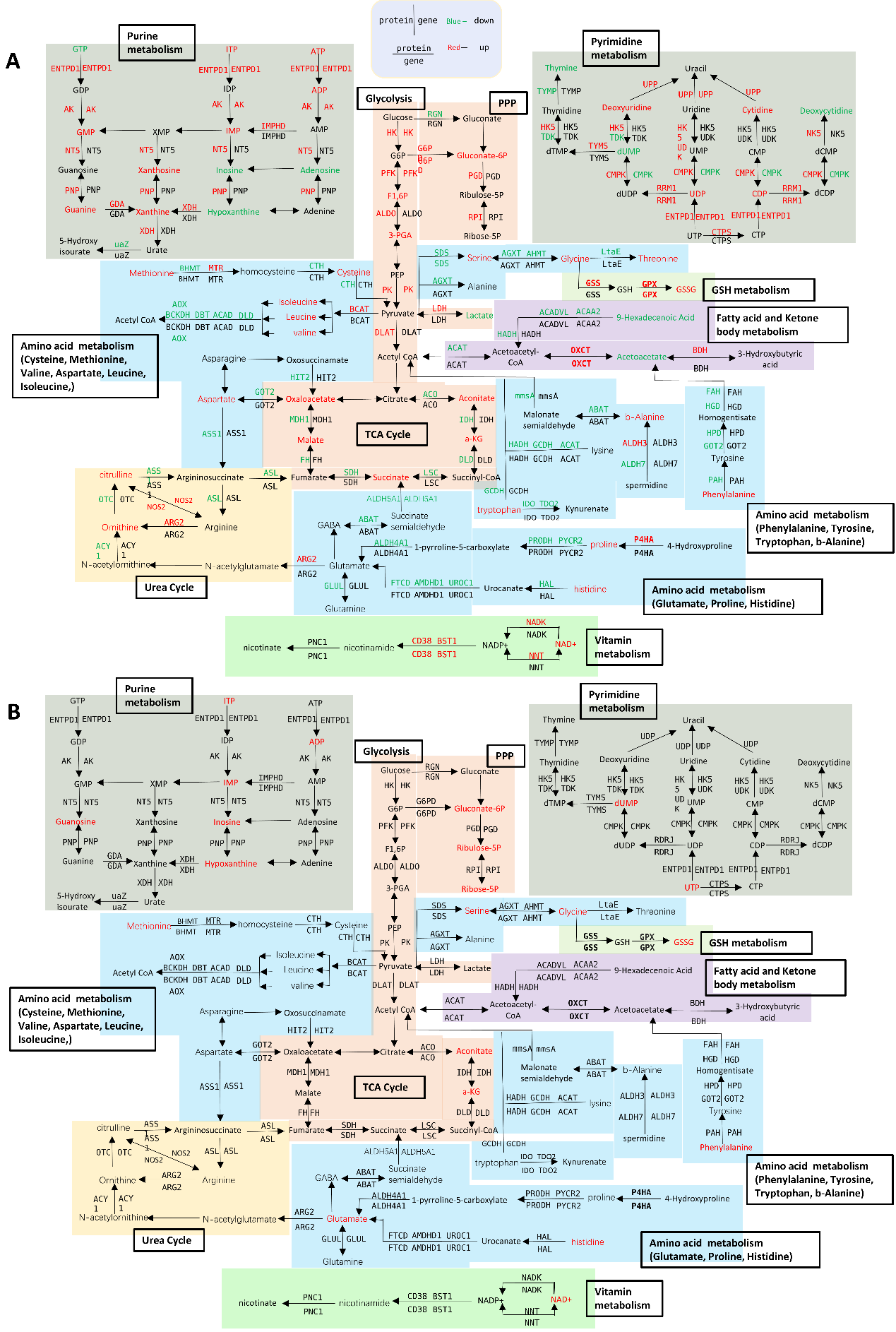


Figure S4. Constructed metabolic pathways that integrated relative metabolite contents, protein and gene expression for two different exposure ways (IV and IG) of mice liver by MSNs-treatment. (A) IV, (B) IG.
